# *Bacteroides fragilis* outer membrane vesicles preferentially activate innate immune receptors compared to their parent bacteria

**DOI:** 10.3389/fimmu.2022.970725

**Published:** 2022-09-20

**Authors:** William J. Gilmore, Ella L. Johnston, Natalie J. Bitto, Lauren Zavan, Neil O'Brien-Simpson, Andrew F. Hill, Maria Kaparakis-Liaskos

**Affiliations:** ^1^ Department of Microbiology, Anatomy, Physiology and Pharmacology, School of Agriculture, Biomedicine and Environment, La Trobe University, Melbourne, VIC, Australia; ^2^ Research Centre for Extracellular Vesicles, School of Agriculture, Biomedicine and Environment, La Trobe University, Melbourne, VIC, Australia; ^3^ ACTV Research Group, Centre for Oral Health Research, Royal Dental Hospital, Melbourne Dental School, The University of Melbourne, Melbourne, VIC, Australia; ^4^ Department of Biochemistry and Chemistry, School of Agriculture, Biomedicine and Environment, La Trobe University, Melbourne, VIC, Australia; ^5^ Institute for Health and Sport, Victoria University, Melbourne, VIC, Australia

**Keywords:** bacterial membrane vesicles, outer membrane vesicles (OMVs), *Bacteroides fragilis*, TLRs, NOD1, innate immunity, epithelial cells, Commensals

## Abstract

The release of bacterial membrane vesicles (BMVs) has become recognized as a key mechanism used by both pathogenic and commensal bacteria to activate innate immune responses in the host and mediate immunity. Outer membrane vesicles (OMVs) produced by Gram-negative bacteria can harbor various immunogenic cargo that includes proteins, nucleic acids and peptidoglycan, and the composition of OMVs strongly influences their ability to activate host innate immune receptors. Although various Gram-negative pathogens can produce OMVs that are enriched in immunogenic cargo compared to their parent bacteria, the ability of OMVs produced by commensal organisms to be enriched with immunostimulatory contents is only recently becoming known. In this study, we investigated the cargo associated with OMVs produced by the intestinal commensal *Bacteroides fragilis* and determined their ability to activate host innate immune receptors. Analysis of *B. fragilis* OMVs revealed that they packaged various biological cargo including proteins, DNA, RNA, lipopolysaccharides (LPS) and peptidoglycan, and that this cargo could be enriched in OMVs compared to their parent bacteria. We visualized the entry of *B. fragilis* OMVs into intestinal epithelial cells, in addition to the ability of *B. fragilis* OMVs to transport bacterial RNA and peptidoglycan cargo into Caco-2 epithelial cells. Using HEK-Blue reporter cell lines, we identified that *B. fragilis* OMVs could activate host Toll-like receptors (TLR)-2, TLR4, TLR7 and nucleotide-binding oligomerization domain-containing protein 1 (NOD1), whereas *B. fragilis* bacteria could only induce the activation of TLR2. Overall, our data demonstrates that *B. fragilis* OMVs activate a broader range of host innate immune receptors compared to their parent bacteria due to their enrichment of biological cargo and their ability to transport this cargo directly into host epithelial cells. These findings indicate that the secretion of OMVs by *B. fragilis* may facilitate immune crosstalk with host epithelial cells at the gastrointestinal surface and suggests that OMVs produced by commensal bacteria may preferentially activate host innate immune receptors at the mucosal gastrointestinal tract.

## Introduction

Bacterial membrane vesicles (BMVs) are nanoparticles released by both pathogenic and non-pathogenic bacteria as part of their normal growth. BMVs are referred to as outer membrane vesicles (OMVs) or membrane vesicles (MVs), if produced by Gram-negative or Gram-positive bacteria, respectively. BMVs contain a range of biological and immunogenic cargo originating from their parent bacteria which includes proteins (1), DNA (2), RNA (3), lipids (4) and peptidoglycan (5, 6), and also lipopolysaccharides (LPS) if produced by Gram-negative bacteria (7). In addition to containing a range of biological cargo, pathogen-derived BMVs can also harbor virulence effectors and immunostimulatory molecules derived from their parent bacteria, enabling them to enhance pathogenesis in the host (8). Due to the diverse range of biological cargo associated with BMVs, they can activate a wide range of host pattern recognition receptors (PRRs) which include Toll-like receptors (TLRs) at the host cell surface, or they can enter host cells and deliver their cargo to intracellular TLRs or nucleotide-binding oligomerisation domain-containing protein (NOD) receptors to mediate inflammation in the host (8). More recently, it has been demonstrated that BMVs produced by pathogenic bacteria may also be enriched in specific cargo compared to their parent bacteria which can include toxins (9), proteins (7), LPS (7), peptidoglycan (6), lipids (4), DNA (10), and RNA (11), and that the differential enrichment of cargo into pathogen-derived BMVs can enhance their immunostimulatory or immunomodulatory functions (12). Therefore, pathogen-derived BMVs are immunogenic, and can be enriched in cargo that facilitates pathogenesis independently of their parent bacteria.

In addition to pathogen-derived BMVs that can elicit immune responses in the host (8), commensal bacteria and their secreted BMVs can also be immunostimulatory or immunomodulatory in the host (13). Recently, the gut microbiota has emerged as a key player in regulating host immune responses, and one mechanism by which they do this is *via* the secretion of immunomodulatory BMVs (13). A large body of evidence now demonstrates that microbiota-derived BMVs contain diverse bacterial cargo including proteins (14–19), RNA (18, 20) and peptidoglycan (21), and that they can deliver their cargo to host cells to activate PRRs and drive immune responses (22–28). One important member of the gut microbiota is *Bacteroides fragilis*, which constitutes 1-2% of the normal intestinal microflora (29), and has the ability to modulate host immunity by mediating IL-10 production as a result of detection of its polysaccharide A (PSA) capsule by TLR2 (30–32). It also was identified that *B. fragilis* OMVs contain PSA and can modulate host immunity, as a result of TLR2 activation and the secretion of IL-10, to ultimately confer protection against colitis in murine models of disease (22, 25). *B. fragilis* OMVs can also contain proteins and lipid A (15), however, it is unclear whether other immunostimulatory bacterial components such as nucleic acids or peptidoglycan are also present in *B. fragilis* OMVs, and their ability to activate innate immune receptors remains unknown.

In this study, we examined the immunogenic cargo associated with *B. fragilis* OMVs and investigated their ability to enter host epithelial cells and activate PRRs. We showed that *B. fragilis* OMVs contain proteins, DNA, RNA, LPS and peptidoglycan. Additionally, we identified that *B. fragilis* OMVs could enter host intestinal epithelial cells and transport their peptidoglycan and RNA cargo intracellularly, rendering this cargo accessible to intracellular PRRs. Moreover, due to the cargo they packaged and their ability to enter host cells, we identified that *B. fragilis* OMVs were able to activate cell-surface receptors TLR2 and TLR4, as well as intracellular PRRs TLR7 and NOD1, in a dose-dependent manner. In comparison, *B. fragilis* bacteria were only able to activate TLR2 and did not activate any other PRRs examined. Collectively, our data demonstrates that *B. fragilis* OMVs are laden with potentially immunogenic cargo that enables them to activate a broader range of PRRs compared to their parent bacteria. These findings highlight the importance of OMV secretion by the commensal *B. fragilis* in maintaining intercellular communication at the mucosal epithelial cell surface.

## Materials and methods

### Bacterial culturing conditions

*Bacteroides fragilis* strain NCTC 9343 was cultured as previously described (33). Briefly, *B. fragilis* was cultured using Horse Blood Agar medium consisting of Blood Agar Base No. 2 (Oxoid, USA) supplemented with 8% (v/v) horse blood (Australian Ethical Biologicals, Australia), or using Brain Heart Infusion (BHI) broth (BD Biosciences, USA) supplemented with 5 μg/ml Hemin (Sigma-Aldrich, USA) with shaking at 120 rpm. Cultures were grown at 37°C in anaerobic conditions using an AnaeroGen 2.5L sachet (Oxoid, USA) and an AnaeroJar 2.5L anaerobic jar (Oxoid, USA).

### Isolation of *B. fragilis* OMVs

*B. fragilis* OMVs were isolated using our established methods of OMV isolation (5, 6, 12, 34–36). Briefly, BHI broth was inoculated using an overnight *B. fragilis* culture at a starting optical density (O.D. _600 nm_) of 0.05 and grown at 37°C with shaking for 16 h to stationary phase of growth (O.D. _600 nm_ of approximately 1.8-2.0) using anaerobic conditions. Bacteria were pelleted by centrifugation at 3, 800 × g for 1 h at 4°C, and the supernatant was subsequently filtered using a 0.22μm polyethersulfone (PES) filter (Nalgene, USA) to remove any remaining bacteria. OMVs contained within bacterial free supernatants were concentrated by tangential flow filtration using a VivaFlow 200 PES crossflow cassette with a 10 kDa molecular weight cut-off filter (Sartorius, Australia), and then pelleted by ultracentrifugation at 100, 000 × g for 2 h at 4°C using a P28S rotor in a CP100NX ultracentrifuge (Hitachi, Japan). The resulting OMV pellets were resuspended in Dulbecco’s phosphate-buffered saline (DPBS; Gibco, USA) and stored at -80°C until further purified.

### Purification of *B. fragilis* OMVs

*B. fragilis* OMVs were purified by OptiPrep (60% iodixanol(v/v); Sigma-Aldrich, USA) density gradient ultracentrifugation as previously described (6, 12, 35, 36). In brief, OMV samples were adjusted to 45% (v/v) OptiPrep in 2ml DPBS and were then overlaid with a discontinuous OptiPrep gradient containing 2ml each of 40%, 35%, 30%, 25% and 20% OptiPrep (v/v) in DPBS. The OptiPrep gradient was subjected to ultracentrifugation at 100, 000 × g for 16 h at 4°C. Twelve fractions (1 ml each) were collected, each fraction was washed with 10 volumes of DPBS by ultracentrifugation at 100, 000 × g for 2 h at 4°C, and then resuspended in DPBS. Fractions 3 to 9 containing purified OMVs were pooled and washed using ultracentrifugation at 100,000 × g for 2 h at 4°C and the purity of OMV preparations was confirmed using Transmission electron microscopy (TEM). Purified OMVs were stored at -80°C until required.

### Nanoparticle tracking analysis (NTA)

Quantification of purified OMVs was performed using ZetaView™ Nanoparticle Tracking Analysis (NTA; Particle Metrix, Germany) as previously described (6). Briefly, OMVs were diluted in DPBS to a concentration of 50 - 200 particles per field of view. NTA measurements of OMV samples were performed using a 488 nm 40 mW laser and CMOS camera by observing 11 cell positions at 25°C with 60 frames captured per position. Analysis was then performed using ZetaView software version 8.05.14 SP7 (minimum brightness: 30, maximum brightness: 255, minimum area: 5, maximum area: 1000, minimum trace length: 15). The average of three biological replicates was calculated and plotted as particle size versus particles per ml using GraphPad Prism v9.3.1.

### Transmission electron microscopy (TEM)

TEM sample preparation was performed as previously described (5, 35). Briefly, OMVs were coated onto carbon‐coated 400 mesh copper grids (ProSciTech, Australia) for 10 min, fixed in 1% (w/v) glutaraldehyde (Sigma-Aldrich, USA) and negatively-stained with 2% (w/v) uranyl-acetate (ProSciTech, Australia). OMV samples were then coated with 2% (w/v) methyl-cellulose (Sigma-Aldrich, USA) in 0.4% (w/v) uranyl acetate. Samples were air dried and viewed using a JEM-2100 transmission electron microscope (JEOL, Japan) operated at 200 kV using a Valeta 4 MP CCD camera (Emsis, Germany).

### Quantification of the cargo associated with *B. fragilis* OMVs and *B. fragilis* bacteria

The protein cargo associated with *B. fragilis* OMVs was quantified using Qubit protein assay (Invitrogen, USA) using a Qubit 3.0 Fluorometer, according to the manufacturer’s instructions.

OMV-associated DNA was quantified using Qubit broad-range DNA assay. Briefly, OMVs were incubated with 4U Turbo DNase (Invitrogen, USA) at 37°C for 1 h to degrade extra-vesicular DNA, according to the manufacturer’s rigorous DNA degradation protocol. Alternatively, OMVs were incubated at 37°C for 1 h with DPBS as a control. To confirm DNase activity, *B. fragilis* genomic DNA was extracted using the Wizard Genomic DNA Purification Kit (Promega, USA) and incubated with DNase as a control. DNA associated with OMVs and controls was quantified using a Qubit 3.0 Fluorometer, according to the manufacturer’s instructions.

OMV associated RNA was quantified using the Qubit high-sensitivity assay. Briefly, OMVs were incubated with 10 pg/μl RNase A (Invitrogen, USA) at 37°C for 1 h as previously described (37), or OMVs were incubated with DPBS as a control. To confirm the efficiency of RNase, *B. fragilis* RNA was extracted using the Isolate II RNA Mini Kit (Bioline, UK) and incubated with RNase as a control. RNA in samples was quantified using a Qubit 3.0 Fluorometer, according to the manufacturer’s instructions.

OMV-associated peptidoglycan was quantified as described previously (5, 6, 38). Briefly, OMV samples and L-18 muramyldipeptide (MDP) standards (Invivogen, USA) were adjusted to a volume of 0.5 ml in 1M NaOH and incubated at 38°C for 30 min. Samples were then incubated with 0.5 ml of 0.5M H_2_SO_4_ and 5 ml concentrated H_2_SO_4_ at 95°C for 5 min. Samples were cooled immediately under running water, followed by the addition of 50 μl CuSO_4_ (4% w/v) and 100 μl of 1.5% (w/v) 4-phenylphenol (dissolved in 96% (v/v) ethanol) and incubated at 30°C for 30 min. Absorbance was measured at 560 nm using a spectrophotometer and the amount of peptidoglycan associated with *B. fragilis* OMVs was determined using the MDP standard curve.

LPS associated with OMVs was quantified using the Pierce Chromogenic Endotoxin Quant kit, according to the manufacturer’s instructions (Thermo Scientific, USA). Briefly, OMV samples and LPS standards were adjusted to a volume of 50 μl and incubated with 50 μl limulus amoebocyte lysate for 9 min. Chromogenic substrate solution was added and samples were incubated for 6 min at 37°C, then the reaction was stopped by the addition of 25% (v/v) acetic acid. Absorbance was measured at 405 nm using a CLARIOstar plate reader (BMG Labtech, Germany) and the amount of LPS associated with 10^8^ OMVs was quantified using the standard curve (0.1-1.0 EU/ml), according to the manufacturer’s instructions. Each assay was performed in technical triplicate.

### Detection of protein, LPS and peptidoglycan cargo associated with *B. fragilis* OMVs and *B. fragilis* bacteria

To detect proteins associated with either *B. fragilis* OMVs or *B. fragilis* bacteria, samples were boiled at 95°C for 5 min in 1x NuPAGE LDS sample buffer (Invitrogen, USA) and 1x NuPAGE Reducing Agent (Invitrogen, USA). Samples were normalized by an equivalent amount of protein and were separated by SDS-PAGE as previously described (6). Proteins associated with *B. fragilis* OMVs and *B. fragilis* bacteria were detected by staining SDS-PAGE gels using Sypro Ruby (Invitrogen, USA), according to the manufacturer’s instructions, and visualized at 560 nm using a ChemiDoc image system (Bio-Rad Laboratories, USA).

Peptidoglycan associated with *B. fragilis* OMVs and their parent bacteria was detected by Western immunoblot, as described previously (6). In brief, 10 μg of *B. fragilis* OMVs and *B. fragilis* bacterial samples were separated by SDS-PAGE, transferred to a 0.2 μm polyvinylidene difluoride membrane and then blocked using 5% (w/v) bovine serum albumin (BSA; Sigma-Aldrich, USA) in Tris-buffered saline containing 0.05% (v/v) Tween (TBS-T). The membrane was then incubated with an anti-peptidoglycan mouse monoclonal antibody (Bio-Rad Laboratories, USA; clone number 3F6B3, 1:1,000 dilution), washed and then incubated with goat anti-mouse IgG HRP antibody (Invitrogen, USA, 1:5,000 dilution). The membranes were then washed, developed using Clarity Western ECL Substrate (Bio-Rad Laboratories, USA) and imaged using a GE Amersham imager 600 (GE Life Sciences, UK).

To detect LPS associated with *B. fragilis* OMVs and their parent bacteria, samples (10 μg protein) were first incubated with proteinase K (10 μM; Invitrogen, USA), or DPBS as a control, for 90 min at 37°C. Samples were then resuspended in 1x NuPAGE LDS sample buffer and 1x NuPAGE Reducing Agent and separated by SDS-PAGE. Next, to visualize LPS, SDS-PAGE gels were stained using ProQ Emerald 300 LPS stain kit (Invitrogen, USA), according to the manufacturer’s instructions. Briefly, samples were fixed [50% methanol (v/v), 5% acetic acid (v/v)] for 90 min, oxidized using periodic acid containing 3% acetic acid (v/v) for 30 min, and stained with ProQ Emerald stain for 2 h. SDS-PAGE gels were then washed using 3% acetic acid (v/v), and LPS was visualized at 300 nm using a ChemiDoc image system (Bio-Rad Laboratories). To determine the amount of protein associated with these samples, SDS-PAGE gels were then counterstained using Sypro Ruby protein stain and visualized at 560 nm.

### Cell culture and stimulations

Human intestinal epithelial cells (Caco-2) were routinely cultured as previously described (39). In brief, Caco-2 cells were cultured in high-glucose Dulbecco’s modified eagle medium (DMEM; Gibco, USA) supplemented with 10% (v/v) fetal calf serum (FCS; Gibco, USA), 1% (v/v) L-glutamine (Gibco, USA), 1% (v/v) penicillin-streptomycin (Gibco, USA), 1% non-essential amino acids (Gibco, USA) and 25mM HEPES (Gibco, USA). HEK-Blue null cells and HEK-Blue hTLR2, hTLR4, hTLR7, hTLR8, hTLR9, hNOD1 and hNOD2 cells (Invivogen, USA) were maintained in DMEM supplemented with 10% (v/v) FCS, 1% (v/v) L-glutamine, 1% (v/v) penicillin-streptomycin and selective antibiotics required for each individual cell line as described previously (6, 40). All cell lines were cultured at 37°C with 5% CO_2_.

To perform HEK-Blue assays, HEK-Blue cells were seeded in 96-well plates (Greiner, Germany) at a density of 1 × 10^5^ cells per well in 200 μl culture media and cultured to approximately 80-90% confluence. *B. fragilis* bacteria were cultured for 16 h and washed with PBS, then added to HEK-Blue cells at an increasing multiplicity of infection (MOI) for 18 h. Alternatively, *B. fragilis* bacteria were heat-killed at 95°C for 45 min as previously described (41), before their addition to HEK-Blue cells at an increasing MOI for 18 h. Viable counts were performed on live and heat-killed *B. fragilis* bacteria by enumerating serial dilutions spread on horse blood agar and cultured overnight at 37°C using anaerobic conditions. Additionally, HEK-Blue cells were either stimulated with an increasing MOI of purified *B. fragilis* OMVs for 18 h, or not-stimulated as negative controls. Positive controls for each cell line included: 50 ng/ml Pam3CSK4 (Pam3CysSerLys4) for TLR2 cells (Invivogen, USA), 6.25 ng/ml LPS for TLR4 cells (Invivogen, USA), 1 pg/ml R848 (resiquimod) for TLR7 and TLR8 cells (Invivogen, USA), 5 nM CpG ODN for TLR9 cells (Invivogen, USA), 100ng/ml TriDap for NOD1 cells (Invivogen, USA) and 0.001 pg/ml L18‐MDP for NOD2 cells (Invivogen, USA). After 24 h, 20 μl of cell culture supernatant was transferred to a fresh 96-well plate and incubated with 180 μl of QUANTI-Blue solution (Invivogen, USA) at 37°C. SEAP activity was measured at 625 nm using a CLARIOstar plate reader (BMG Labtech, Germany).

### MTT cell viability assay

The viability of HEK-Blue cells following 18 h stimulation with either live or heat-killed bacteria, or not-stimulated as controls, was determined using the MTT Cell Proliferation Kit (Abcam, UK), according to the manufacturer’s protocol. Briefly, HEK-Blue cell lines (Null, hTLR2, hTLR4, hTLR7 and hNOD1) were seeded at 1 × 10^5^ cells per well in 96-well plates and stimulated with either live or heat-killed *B. fragilis* bacteria at an MOI of 1,000 for 18 h. Culture media was then replaced with DMEM containing 100 μg/ml gentamicin for 2 h. The media was then replaced with 50 μl MTT reagent in 50 μl DMEM for 3 h followed by adding 150 μl MTT solvent for 15 minutes with shaking. Absorbance was measured at 590 nm using a CLARIOstar plate reader (BMG Labtech, Germany).

### Fluorescent labelling of OMVs and OMV-associated cargo

*B. fragilis* OMVs were labelled using Vybrant DiI (10 μM; Invitrogen, USA) as described previously (6, 10, 42–44). Briefly, OMVs were adjusted to 1.2 × 10^12^ OMVs per ml in 100 μl of DPBS and stained with DiI for 30 min at 37°C with gentle agitation. The RNA content of OMVs was labelled by incubating with Syto RNASelect (1μM; Invitrogen, USA) for 60 min with gentle agitation, as previously described (43–45). The peptidoglycan content of *B. fragilis* OMVs was labelled using BODIPY-FL vancomycin (4 ng/ml; Invitrogen, USA) and non-labelled vancomycin (4 ng/ml; Sigma-Aldrich, USA) for 20 min, as previously described (42). An equivalent amount of each fluorescent stain in DPBS (in the absence of OMVs) was used as a negative control. Excess DiI, Syto RNASelect or BODIPY-FL vancomycin dye were removed by washing OMVs and controls four times with 4 ml DPBS using a 10 kDa centrifugal filtration unit (Merck Millipore, Germany).

### Examining OMV entry into host cells by confocal microscopy

To visualise OMV entry into host cells, Caco-2 cells were seeded on 18mm round coverslips (Marienfeld, Germany) in 12-well plates at a density of 3 × 10^5^ cells per well in 1ml of media for 24 h. Caco-2 cells were stimulated with either DiI, BODIPY-FL or Syto RNASelect-labelled *B. fragilis* OMVs for 4 h at an MOI of 4 × 10^5^ OMVs per cell, or each respective stain in DPBS as a control. Following incubation, cells were washed three times with DPBS, and extracellular fluorescence was quenched with 0.025% (v/v) Trypan blue as previously described (34). Cells were fixed using 4% paraformaldehyde (Sigma-Aldrich, USA) and blocked using 1% BSA (w/v) in DPBS. Cell nuclei and cellular actin were stained with 4’,6‐diamidino‐2‐phenylindole dilactate (DAPI; Merck, Germany) and Alexa Fluor 680 phalloidin (Invitrogen, USA), respectively. Samples were then mounted using VectaShield mounting medium (Vector Laboratories, USA) and imaged using a Zeiss 780 PicoQuant confocal microscope (Zeiss, Germany) using a 63x/1.4NA oil objective at 1024 × 1024 × 32 bit per channel. Image analysis was performed using Imaris x64 v9.5.0 (Bitplane, Switzerland). Three biological replicates of Caco-2 cells stimulated with OMVs, labelled with each individual stain, were examined. Three fields of view were imaged for each treatment containing a minimum of 10 cells per field of view.

### Statistical analysis

All statistical analyses were performed using GraphPad Prism software v9.3.1. Qubit quantification experiments were analysed using an unpaired *t*-test. HEK-Blue experiments were analysed using an unpaired *t*-test or one-way ANOVA with Dunnett’s multiple comparisons test, as indicated. Differences were considered statistically significant when *p < 0.05, **p < 0.01, ***p < 0.001, ****p < 0.0001.

## Results

### *B. fragilis* OMVs contain DNA, RNA, protein, LPS, and peptidoglycan

**Figure 1:**
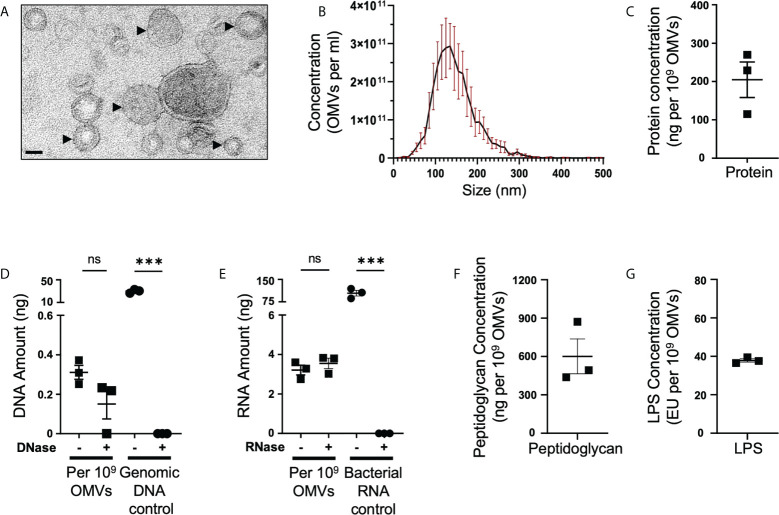
*B fragilis* OMVs are heterogenous in size and morphology, and harbour protein, DNA, RNA, peptidoglycan and LPS cargo. **(A)** Purified *B. fragilis* OMVs were visualized using transmission electron microscopy (TEM). OMVs are indicated by black arrows. Images are representative of three biological replicates (Scale bar = 100 nm). **(B)** The size distribution of *B. fragilis* OMVs was determined using ZetaView Nanoparticle Tracking Analysis. Data shows the mean (black line) ± SEM (red error bars) of three biological replicates. **(C)** Quantification of OMV-associated protein, per 10^9^ *B. fragilis* OMVs, using Qubit fluorometric analysis. Data shows the mean ± SEM of three biological replicates. **(D)** Quantification of DNA associated with DNase-treated (+) or non-treated (-) *B. fragilis* OMVs using Qubit fluorometric analysis. *B. fragilis* genomic DNA (Genomic DNA control), either treated with DNase (+) or non-treated (-), was used as a control for DNase activity. Shown is the mean ± SEM of three biological replicates. ns, not significant, ***p < 0.001 (unpaired *t-*test). **(E)** Quantification of RNA associated with RNase-treated (+) or non-treated (-) *B. fragilis* OMVs using Qubit fluorometric analysis. *B. fragilis* bacterial RNA (Bacterial RNA control), either treated with RNase (+) or not-treated (-), was used as a control for RNase activity. Shown is the mean ± SEM of three biological replicates. ns, not significant, ***p < 0.001 (unpaired *t-*test). **(F)** Quantification of the peptidoglycan cargo associated with 10^9^ *B. fragilis* OMVs. Shown is the mean ± SEM of three biological replicates. **(G)** LPS associated with 10^9^ *B. fragilis* OMVs was quantified using the Pierce™ Chromogenic Endotoxin Quant kit. Data represents the mean ± SEM of three biological replicates.

*B. fragilis* OMVs contain a range of cargo including polysaccharides (22), proteins (15, 16, 46) and LPS (15). However, it remains unclear if *B. fragilis* OMVs also contain other biological cargo including peptidoglycan and nucleic acids. *B. fragilis* OMVs were isolated and purified to examine their biological cargo composition, as well as their size and morphology. Examination of purified *B. fragilis* OMVs using transmission electron microscopy (TEM) and Nanoparticle Tracking Analysis (NTA) revealed that they were heterogeneous in size, with the predominant population of OMVs being approximately 135 nm in diameter (**Figures 1A, B**). Next, we quantified the amount of protein, DNA, RNA, peptidoglycan and LPS cargo associated with *B. fragilis* OMVs (**Figures 1C–G**). Having previously shown that there is variability in the quantification of OMV-associated cargo based on the type of protein assay used and the method used to normalise BMV number (35), we quantified the biological cargo associated with OMVs and represented the quantity of cargo per 10^9^ OMVs (**Figures 1C–G**). Quantification of the cargo associated with 10^9^ *B. fragilis* OMVs revealed that they contained protein, DNA and RNA (**Figures 1C–E**). Furthermore, degradation of extra-vesicular DNA using DNase, or RNA using RNase, revealed that the majority of DNA and RNA was protected from degradation and was therefore predominantly located within OMVs (**Figures 1D, E**). We also identified that *B. fragilis* OMVs contained peptidoglycan, with approximately 600 ng of peptidoglycan associated with 10^9^ OMVs (**Figure 1F**), in addition to LPS with approximately 38 endotoxin units (EU) of LPS per 10^9^ OMVs (**Figure 1G**). Collectively, these findings demonstrate that *B. fragilis* OMVs contain a diverse range of biological cargo including protein, DNA, RNA, peptidoglycan and LPS.

### *B. fragilis* OMVs have an altered cargo composition compared to their parent bacteria

**Figure 2:**
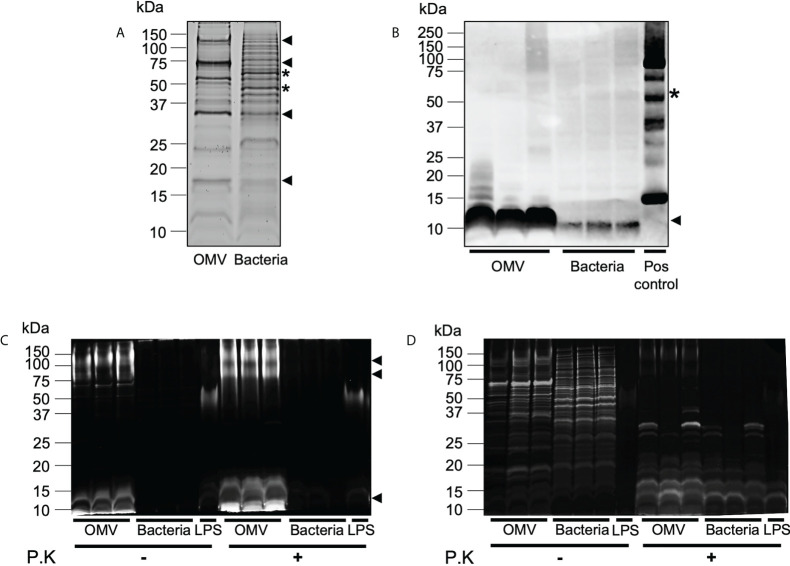
*B. fragilis* OMVs are enriched in protein, peptidoglycan and LPS cargo compared to their parent bacteria. **(A)** *B. fragilis* OMVs (10 μg) and *B. fragilis* bacteria (10 μg) were separated using SDS-PAGE and their protein cargo was stained using Sypro Ruby. Data is representative of three biological replicates. **(B)** The presence of peptidoglycan cargo associated with *B. fragilis* OMVs (10 μg) and *B. fragilis* bacteria (10 μg) was detected using Western immunoblot using an anti-peptidoglycan antibody. Peptidoglycan was used as a positive control (Pos control). Data shows three biological samples of *B. fragilis* OMVs and *B. fragilis* bacteria and is representative of three experiments. **(C)** *B. fragilis* OMVs and *B. fragilis* bacteria were treated with Proteinase K (P.K) (+) or not treated as controls (-), and their LPS cargo was detected by staining with ProQ Emerald 300 LPS stain. **(D)** ProQ Emerald-stained SDS-PAGE gels were counterstained with the protein-specific stain Sypro Ruby. Data in panel C and D represents three independent biological samples of *B. fragilis* OMVs and *B. fragilis* bacteria and is representative of three independent experiments. In all panels **(A–D)**, black arrows represent cargo that is enriched in OMVs compared to bacteria, stars represent cargo that is less abundant in OMVs compared to bacteria.

We have previously demonstrated that BMVs produced by Gram-negative and Gram-positive bacteria contain cargo that differs from their parent bacterium, suggesting that bacteria can preferentially package or enrich biological cargo in their BMVs (6, 12). Furthermore, we showed that enrichment of biological cargo within BMVs can have a profound effect on their subsequent biological functions, when compared to their parent bacteria and to BMVs produced during different stages of bacterial growth, indicating that selective cargo packaging into BMVs regulates their functions (12). As *B. fragilis* OMVs contain protein, DNA, RNA, LPS and peptidoglycan, we next investigated if *B. fragilis* OMVs were enriched in biological cargo compared to their parent bacteria. Examination of the overall protein profiles of *B. fragilis* OMVs by SDS-PAGE revealed that a range of predominant protein bands, including those at approximately 125 kDa, 75 kDa, 30 kDa and 17 kDa, were enriched in *B. fragilis* OMVs compared to their parent bacteria (**Figure 2A**, arrows). Similarly, some protein bands present in *B. fragilis* bacteria, such as bands of approximately 60 and 45 kDa, were not equally prominent in OMVs (**Figure 2A**, stars), suggesting that there is selective cargo packaging of protein into *B. fragilis* OMVs.

We next detected the presence of peptidoglycan within OMVs and bacterial samples by Western immunoblot. Examination of an equivalent amount of *B. fragilis* OMVs and bacteria, normalized by protein amount, revealed that OMVs were enriched in peptidoglycan, evidenced by a prominent band of approximately 10 kDa, compared to their parent bacteria (**Figure 2B**). These differences in peptidoglycan profiles suggest differences in peptidoglycan packaging into OMVs compared to their parent bacteria, similar to what we have previously observed in MVs produced by the Gram-positive pathogen *S. aureus* (6).

Although it was previously reported that there were no structural differences between the lipid A LPS moiety of *B. fragilis* and their OMVs (15), the relative abundance of LPS cargo in *B. fragilis* OMVs compared to their parent bacteria remains unknown. To address this, we examined the LPS profiles of *B. fragilis* OMVs compared to their parent bacteria using SDS-PAGE, loaded with equivalent protein amounts, and visualized LPS using the ProQ Emerald stain (**Figure 2C**). LPS moieties of approximately 100 kDa, 75 kDa and 10 kDa were prominent in *B. fragilis* OMVs, however these moieties were less evident in *B. fragilis* bacterial samples, suggesting that LPS was enriched in OMVs compared to their parent bacteria (**Figure 2C**). The higher molecular weight staining (approximately 75 to 100 kDa) in the *B. fragilis* OMV samples suggests the presence of LPS. In contrast, the low molecular weight smear (10 kDa) may represent lipooligosaccharide (LOS) or lipid A components (**Figure 2C**), as described previously (40). Additionally, both high and low molecular weight smears were resistant to proteinase K degradation (**Figure 2C**) and were not visualized by Sypro Ruby staining (**Figure 2D**), validating that these bands were representative of LPS. Taken together, these data demonstrate that *B. fragilis* OMVs harbor a range of biological cargo that includes proteins, peptidoglycan, and LPS and that there are differences in the preferential packaging of these immunostimulatory cargo into OMVs compared to their parent bacteria, which may ultimately alter their ability to activate host PRRs.

### *B. fragilis* OMVs activate TLR2 and TLR4 responses compared to their parent bacteria that can only activate TLR2

**Figure 3:**
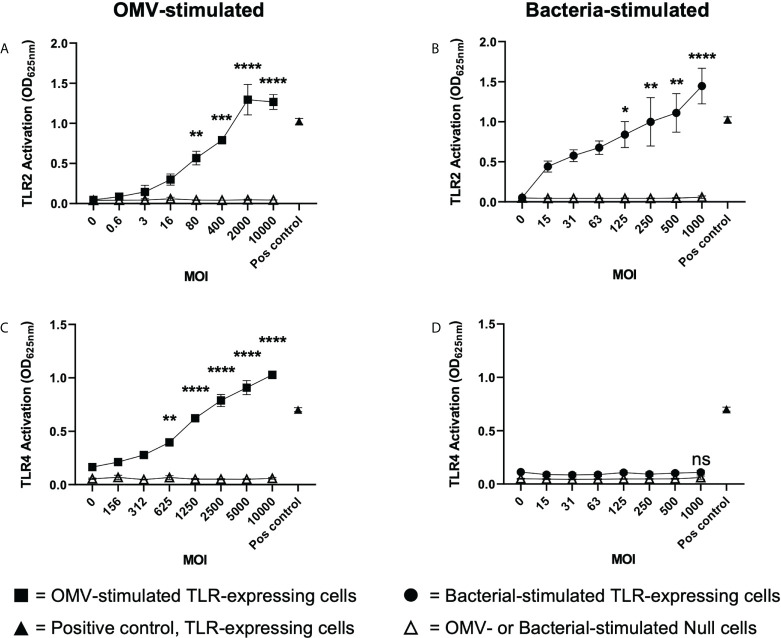
*B. fragilis* OMVs activate TLR2 and TLR4 compared to their parent bacteria which only activate TLR2. **(A, B)** TLR2 and **(C, D)** TLR4 expressing HEK-Blue cells were stimulated with either *B. fragilis* OMVs (**A**, **C**, squares) or *B fragilis* bacteria (**B**, **D**, circles) at an increasing multiplicity of infection (MOI) for 18 hours. In all panels, open triangles indicate stimulation of the HEK-Blue null cell line with either OMVs **(A, C)** or bacteria **(B, D)** in each assay, filled triangles indicate positive controls for each respective cell line. Data represents mean ± SEM of three biological replicates. ns = not significant, *p < 0.05, **p < 0.01, ***p < 0.001, ****p < 0.0001 (One-way ANOVA with Dunnett’s multiple comparisons test, compared to non-stimulated controls).

Having revealed that *B. fragilis* OMVs contain various biological cargo with distinct differences in their cargo composition compared to their parent bacteria (**Figures 2A–D**), we sought to determine if there was a difference in the ability of *B. fragilis* OMVs and their parent bacteria to activate host PRRs. To address this, HEK-Blue reporter cells that express either human TLR2 or TLR4, or control HEK-Blue null cells, were stimulated with an increasing dose of either *B. fragilis* OMVs, live *B. fragilis* bacteria, or heat killed *B. fragilis* bacteria as a control. First, we identified that *B. fragilis* OMVs as well as live or heat-killed bacteria were unable to induce the activation of HEK-Blue null cells (**Figures 3A–D**; **Supplementary Figure 1A**). However, both *B. fragilis* OMVs and their parent bacteria activated TLR2 in a dose-dependent manner (**Figures 3A, B**), consistent with previous reports (22, 32). OMV-mediated TLR2 activation occurred at an MOI as low as approximately 80 OMVs per cell (p < 0.01), suggesting that OMVs can readily activate TLR2 (**Figure 3A**). Next, we investigated whether *B. fragilis* and their OMVs could activate TLR4, and observed dose-dependent activation of TLR4 by *B. fragilis* OMVs, which occurred at MOI as low as approximately 625 OMVs per cell (p < 0.01) (**Figure 3C**). However, we did not observe any TLR4 activation in response to stimulation with live *B. fragilis* bacteria at any of the concentrations examined, suggesting that there is a difference in the TLR4 immunostimulatory abilities of *B. fragilis* OMVs compared to their parent bacteria (**Figure 3D**). Similarly, heat-killed *B. fragilis* bacteria also activated TLR2 but not TLR4-expressing HEK-Blue cells (**Supplementary Figures 1B, C**), identifying that the inability of *B. fragilis* to activate TLR4 was independent of bacterial viability or potential cytotoxic effects mediated by live bacteria on host cells. We also confirmed the viability of HEK-Blue null cells and TLR2 and TLR4 expressing HEK-Blue cells following stimulation with either live or heat killed *B. fragilis* bacteria at maximal MOI (**Supplementary Figures 1D–F**), validating that the lack of TLR4 activation in response to stimulation with live *B. fragilis* bacteria was not due to a decrease in HEK-Blue cell viability. Overall, these data demonstrate that *B. fragilis* OMVs activate both TLR2 and TLR4, whereas live and heat killed *B. fragilis* bacteria can only activate TLR2, suggesting that the immunogenic cargo of *B. fragilis* OMVs may enhance their ability to activate TLR4 compared to their parent bacteria.

### *B. fragilis* OMVs can enter host intestinal epithelial cells and deliver peptidoglycan and RNA intracellularly

**Figure 4:**
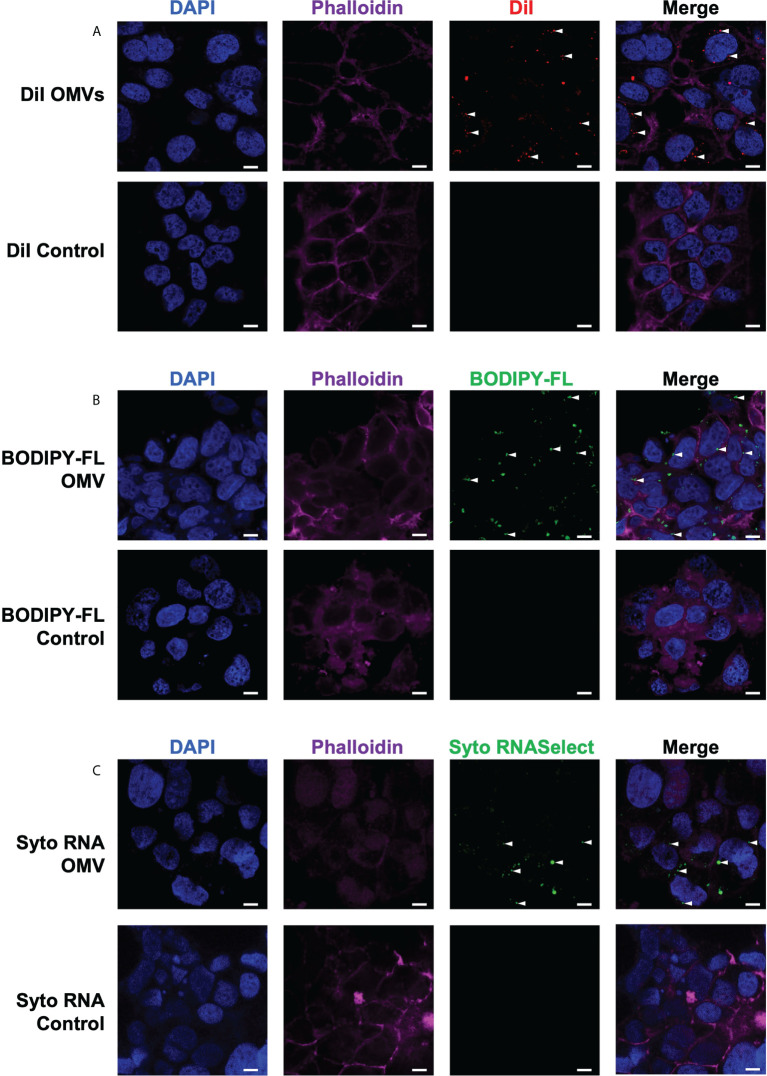
*B fragilis* OMVs enter host intestinal epithelial cells and transport their peptidoglycan and RNA cargo intracellularly. The **(A)** lipid (DiI; red), **(B)** peptidoglycan (BODIPY-FL vancomycin; green) and **(C)** RNA (Syto RNASelect; green) cargo associated with *B. fragilis* OMVs was fluorescently labelled. Fluorescently labelled *B. fragilis* OMVs were then incubated with Caco-2 cells for 4 hours, and OMV entry was visualized by confocal microscopy. Cell nuclei and cellular actin were visualized by staining with DAPI (blue) or Phalloidin (magenta). Intracellular *B. fragilis* OMVs are indicated by white arrows. DPBS containing each respective stain (Control) were incubated with Caco-2 cells to control for the non-specific uptake of each fluorescent stain by host cells. Images are representative of three biological replicates of Caco-2 cells stimulated with each type of fluorescently-labelled *B. fragilis* OMVs. Scale bar = 10 μm.

We next investigated the ability of *B. fragilis* OMVs to enter and deliver their immunogenic cargo, including peptidoglycan and RNA, to host epithelial cells. First, we confirmed the ability of *B. fragilis* OMVs to enter human intestinal epithelial cells (Caco-2). To do this, DiI-labelled *B. fragilis* OMVs were incubated with Caco-2 cells for 4 hours, and the ability of OMVs to enter epithelial cells was determined by confocal microscopy (**Figure 4A**). Examination revealed that *B. fragilis* OMVs were capable of entering Caco-2 epithelial cells, and therefore able to deliver their immunogenic cargo intracellularly to host cells (**Figure 4A**).

We have previously shown that *Helicobacter pylori* OMVs contain peptidoglycan, and that they can deliver their peptidoglycan cargo into host epithelial cells, resulting in the activation of the cytoplasmic host innate immune receptor NOD1 and the induction of an innate immune response (5, 42). As *B. fragilis* OMVs also contain peptidoglycan (**Figures 1**, **2**), we next investigated the ability of *B. fragilis* OMVs to deliver their peptidoglycan cargo intracellularly to host epithelial cells. To do this, peptidoglycan associated with *B. fragilis* OMVs was fluorescently labelled using BODIPY-FL vancomycin, and the ability of fluorescently-labelled *B. fragilis* OMVs to enter Caco-2 cells was determined using confocal microscopy (**Figure 4B**). We found that BODIPY-FL-labelled *B. fragilis* OMVs entered Caco-2 epithelial cells and were therefore capable of delivering their fluorescently-labelled peptidoglycan intracellularly (**Figure 4B**).

Pathogen-derived BMVs can also mediate the intracellular delivery of bacterial RNA to host cells, resulting in the induction of innate immunity (6, 37, 44, 47, 48). However, the ability of commensal-derived BMVs to deliver RNA cargo into host cells remains to be elucidated. Therefore, we next examined the ability of *B. fragilis* OMVs to deliver their RNA cargo intracellularly to intestinal epithelial cells. To do this, RNA associated with *B. fragilis* OMVs was labelled using Syto RNASelect. *B. fragilis* OMVs containing fluorescently-labelled RNA were incubated with Caco-2 cells and examined by confocal microscopy, revealing that *B. fragilis* OMVs could deliver RNA intracellularly to Caco-2 cells (**Figure 4C**). As a control for the non-specific uptake of DiI, BODIPY-FL vancomycin or Syto RNASelect, Caco-2 cells were incubated with each respective stain resuspended in DPBS in the absence of OMVs and examined by confocal microscopy, revealing a lack of each fluorescent stain intracellularly (**Figure 4**, controls). These findings reveal that *B. fragilis* OMVs can enter host epithelial cells to deliver their cargo, that includes peptidoglycan and RNA.

### *B. fragilis* OMVs activate NOD1 and TLR7 responses, whereas their parent bacteria cannot

**Figure 5:**
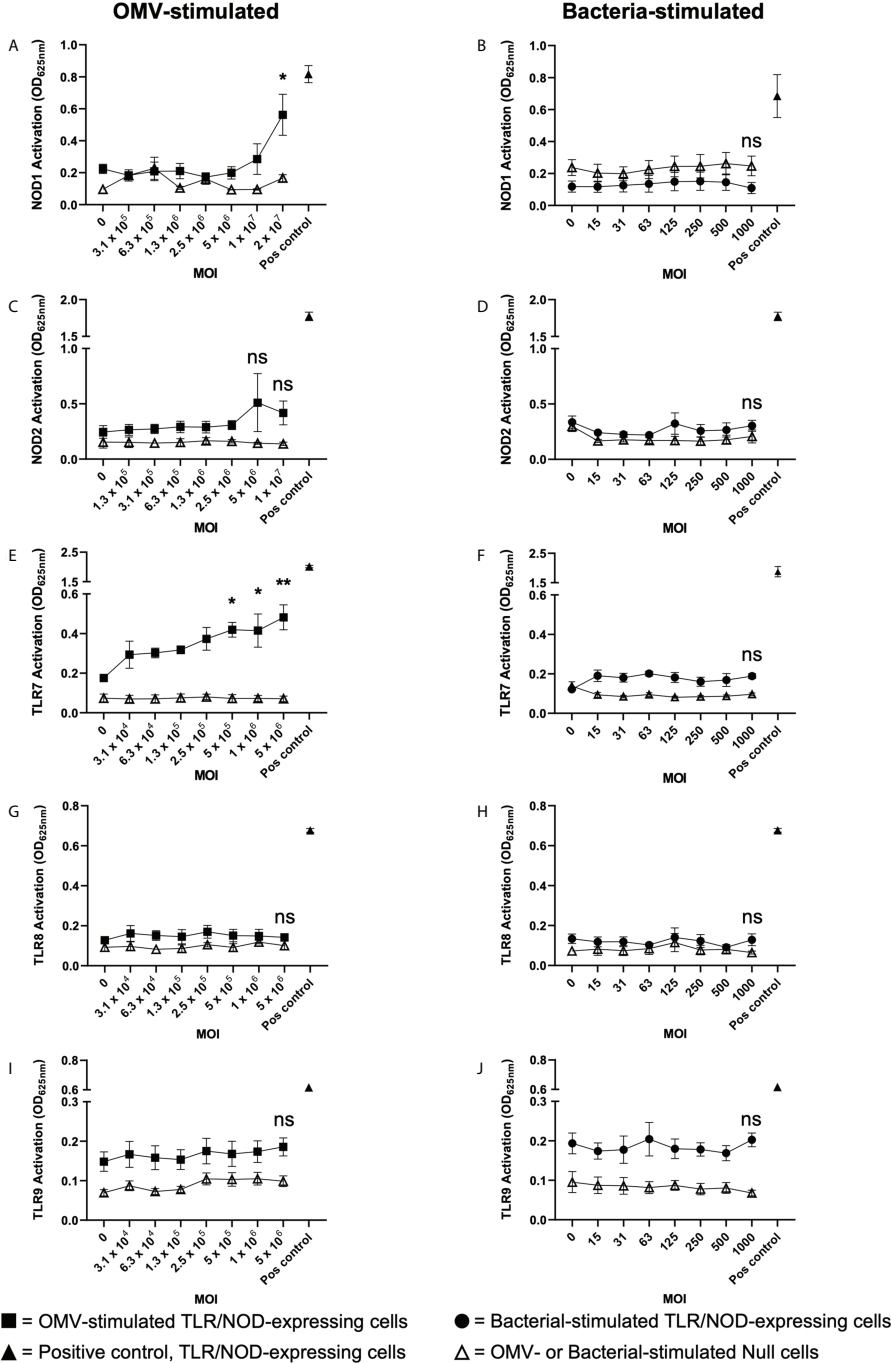
*B fragilis* OMVs activate NOD1 and TLR7 whereas *B fragilis* bacteria cannot. **(A, B)** NOD1, **(C, D)** NOD2, **(E, F)** TLR7, **(G, H)** TLR8 and **(I, J)** TLR9 expressing HEK-Blue cells were stimulated with an increasing MOI of either *B. fragilis* OMVs (**A**, **C**, **E**, **G**, **I**, squares) or *B. fragilis* bacteria (**B**, **D**, **F**, **H**, **J**, circles) for 18 hours. Open triangles indicate stimulation of the HEK-Blue null cell line with either *B. fragilis* OMVs or *B. fragilis* bacteria as a negative control in each assay. Filled triangles indicate positive controls for each respective cell line. Data represents mean ± SEM of three biological replicates. ns=not significant, *p < 0.05, **p < 0.01. (One-way ANOVA with Dunnett’s multiple comparisons test, compared to non-stimulated controls).

We next investigated the ability of *B. fragilis* OMVs to activate the intracellular receptors for peptidoglycan, NOD1 and NOD2, which detect unique components of bacterial peptidoglycan. Specifically, NOD1 detects D-glutamyl-meso-diaminopimelic acid found predominantly in Gram-negative bacteria (49), whereas NOD2 detects muramyl dipeptide found in peptidoglycan of both Gram-negative and Gram-positive bacteria (50). To investigate the immune-stimulating potential of peptidoglycan delivered by *B. fragilis* OMVs, HEK-Blue cells expressing human NOD1 were stimulated with an increasing dose of *B. fragilis* OMVs or *B. fragilis* bacteria (**Figures 5A, B**). We found that stimulation of NOD1-expressing HEK-Blue cells with an MOI of 2 × 10^7^ OMVs per cell resulted in significant activation of NOD1 compared to non-stimulated controls (p < 0.05) (**Figure 5A**). Based on our quantification of peptidoglycan associated with OMVs (**Figure 1D**), this corresponded to approximately 12 ng of peptidoglycan associated with 2 × 10^7^ OMVs resulting in the activation of NOD1 (**Figure 5A**). In comparison, we observed that NOD1 was not activated by *B. fragilis* bacteria at any MOI examined in this study, indicating that there are differences in the ability of *B. fragilis* and their OMVs to activate NOD1 (**Figure 5B**). We next characterized the ability of OMVs to activate NOD2, which has been previously reported to have a role in the detection of *B. fragilis* OMVs by bone marrow-derived dendritic cells (25, 50). However, we did not observe any significant activation of NOD2-expressing HEK-Blue cells by either *B. fragilis* OMVs or *B. fragilis* bacteria at any of MOI examined in this study (**Figures 5C, D**). Furthermore, heat-killed bacteria did not activate either NOD1 or NOD2, and the inability of *B. fragilis* bacteria to activate NOD1 signaling compared to OMVs was not due to a reduction in host cell viability (**Supplementary Figures 2A–C**).

Having determined that *B. fragilis* OMVs harbor both DNA and RNA cargo (**Figures 1D, E**) and that RNA associated with B*. fragilis* OMVs can enter epithelial cells (**Figure 4C**), we next investigated their ability to activate the RNA receptors TLR7 and TLR8, and TLR9 that detects bacterial DNA. To address this, TLR7, TLR8 and TLR9 expressing HEK-Blue cells were stimulated with an increasing dose of *B. fragilis* OMVs or their parent bacteria (**Figures 5E–J**). Stimulation of TLR7 expressing HEK-Blue cells with an increasing dose of *B. fragilis* OMVs resulted in significant activation of TLR7 compared to non-stimulated controls, with a minimum MOI of approximately 5 × 10^5^ OMVs per cell being required to induce TLR7 activation (p < 0.05) (**Figure 5E**). In contrast, live and heat killed *B. fragilis* bacteria could not activate TLR7 at any concentration examined in this study (**Figure 5F**, **Supplementary Figure 3A**). Furthermore, the inability of live or heat killed *B. fragilis* to activate TLR7 compared to OMVs was not due to impairing the viability of HEK-Blue cells expressing TLR7 (**Supplementary Figure 3B**). We also found that neither *B. fragilis* OMVs, nor live or heat-killed *B. fragilis* bacteria were able to induce the activation of TLR8 (**Figures 5G, H**, **Supplementary Figure 3C**), suggesting that *B. fragilis* bacteria were unable to activate either TLR7 or TLR8, and that RNA associated with *B. fragilis* OMVs preferentially activated TLR7 compared to TLR8 (**Figure 5E**). This is in contrast to what we have previously observed using *S. aureus* MVs, where an equivalent amount of RNA-containing *S. aureus* MVs could activate both TLR7 and TLR8 (6). Moreover, we found that live and heat killed *B. fragilis* bacteria and their OMVs could not activate TLR9 responses at all MOIs examined (**Figures 5I, J**, **Supplementary Figure 3D**). The inability of *B. fragilis* OMVs to activate TLR9 responses may be attributed to them having approximately ten-fold less DNA (0.311ng per 10^9^ OMVs) than RNA (3.21ng per 10^9^ OMVs) content (**Figures 1D, E**) and therefore, the amount of DNA delivered by *B. fragilis* OMVs may be insufficient to induce TLR9 activation. Moreover, NF-κB activity was not observed in the negative control HEK-Blue null cell line in response to stimulation with *B. fragilis* OMVs or bacteria, revealing that the delivery of bacterial cargo by *B. fragilis* OMVs is essential for their ability to activate the intracellular receptors NOD1 and TLR7 (**Figure 5**).

Taken together, our data identify the ability of the intestinal commensal *B. fragilis* to package protein, DNA, RNA, peptidoglycan and LPS into their OMVs, and that there is enrichment of immunogenic cargo in *B. fragilis* OMVs. Furthermore, we show the ability of enriched *B. fragilis* OMV-associated cargo to be delivered intracellularly to host cells, which ultimately enables *B. fragilis* OMVs to preferentially activate a broader range of innate immune receptors compared to their parent bacteria. Moreover, these findings identify novel mechanisms of selective immune activation mediated by *B. fragilis* OMVs at the host epithelial cells surface *via* preferential activation of TLR4, TLR7 and NOD1.

## Discussion

The immunostimulatory functions of BMVs depend upon the specific cargo they contain and their ability to deliver this cargo to host cells. BMVs produced by both pathogens and commensals can package various biological cargo including nucleic acids, proteins, LPS and peptidoglycan. Furthermore, pathogen-derived BMVs can be enriched in immunostimulatory cargo, enabling them to activate host PRRs and drive immune responses in the host. However, the enrichment of immunostimulatory cargo in commensal-derived BMVs compared to their parent bacteria, and their subsequent ability to deliver this cargo and activate innate immune receptors is not equally well characterized. The findings of this study reveal that OMVs produced by the commensal *B. fragilis* contain protein, nucleic acids, LPS and peptidoglycan and are enriched in LPS, peptidoglycan and proteins compared to their parent bacterium (**Figures 1**, **2**). Additionally, we show that *B. fragilis* OMVs can enter intestinal epithelial cells to deliver their RNA and peptidoglycan cargo intracellularly (**Figure 4**). Moreover, the enrichment of peptidoglycan, LPS and protein cargo into *B. fragilis* OMVs, in addition to their ability to deliver their cargo into host epithelial cells, enables them to activate a more diverse range of PRRs which includes TLR4, TLR7 and NOD1 compared to their parent bacteria (**Figures 3**, **5**). Collectively, our findings identify the enrichment of select cargo into *B. fragilis* OMVs that subsequently results in their ability to activate a broader range of host innate immune receptors compared to their parent bacteria, revealing that *B. fragilis* OMVs may function to increase the potential for commensal-host crosstalk at the intestinal epithelial barrier.

In this study, we investigated the type and quantity of biological cargo associated with *B. fragilis* OMVs and determined their ability to activate host innate immune receptors compared to their parent bacteria. Our data revealed that *B. fragilis* OMVs contain a wide range of biological cargo consisting of protein, LPS, peptidoglycan, DNA and RNA (**Figure 1**). Interestingly, *B. fragilis* OMVs contained approximately ten-fold more RNA than DNA (**Figures 1D, E**), which is consistent with previous studies identifying that MVs derived from the Gram-positive commensals *Lactobacillus* *reuteri* (20) and *Lactobacillus casei* (18) also packaged significantly more RNA than DNA. While the RNA associated with pathogen-derived BMVs is becoming increasingly recognized as being able to activate innate immune receptors and to modulate cellular functions when delivered into host cells (37, 51), knowledge regarding the immunostimulatory and immunomodulatory abilities of RNA delivered by commensal-derived BMVs is limited, highlighting that future research endeavors should focus on broadening our understanding of their functions.

Our data show that *B. fragilis* OMVs were enriched with protein cargo compared to their parent bacterium (**Figure 2A**). In agreement with our findings, a previous study examining the proteome of *B. fragilis* OMVs found that they were enriched in acidic proteases and sugar-hydrolysing glycosidases, which facilitated the catabolism and acquisition of environmental nutrients and were thought to ameliorate the establishment and composition of the gut microbiota (15). Moreover, while selective protein packaging into pathogen-derived BMVs has been shown to promote pathogen colonisation or survival (52, 53), proteins enriched in BMVs produced by various pathogens also have important roles in facilitating bacterial pathogenesis and promoting the development of inflammatory host immune responses (12, 52–55), further supporting the notion that bacteria can regulate the proteome of their OMVs to modulate their functions.

We also observed the enrichment of LPS into *B. fragilis* OMVs compared to their parent bacteria (**Figure 2C**). Although ProQ Emerald stain used in this study cannot discriminate between different LPS isoforms at the molecular level, the LPS enriched in *B. fragilis* OMVs was characteristic of both larger LPS species, as well as smaller LPS, LOS or lipid A species (**Figure 2C**). Consequently, we observed that *B. fragilis* OMVs, but not their parent bacteria, induced dose-dependent activation of TLR4, the host immune receptor responsible for the detection of LPS. Therefore, our findings suggest that the enrichment of LPS into *B. fragilis* OMVs enhances their ability to activate TLR4 compared to their parent bacteria. In agreement with our findings, a previous study showed that long-chain LPS moieties could be enriched in *Porphyromonas gingivalis* OMVs compared to their parent bacteria (7). Moreover, it was also reported that OMVs produced by wild-type *Neisseria meningitidis* strains induced stronger TLR4 responses compared to OMVs produced by LPS-depleted strains, demonstrating that OMVs containing more LPS were more readily able to induce the activation of TLR4-mediated immune responses (56). Collectively, these studies support our findings that LPS can be enriched in *B. fragilis* OMVs which may contribute to their enhanced capacity to mediate TLR4 signaling compared to their parent bacteria.

In addition to examining the ability of *B. fragilis* OMVs to preferentially activate host cell surface expressed TLRs, we also investigated the ability of *B. fragilis* OMVs to enter epithelial cells, rendering their cargo accessible to intracellular PRRs, and their subsequent ability to activate these cytoplasmic PRRs. Using previously validated methods to label BMVs and their associated peptidoglycan and RNA cargo, we demonstrated that *B. fragilis* OMVs entered intestinal epithelial cells and delivered their fluorescently-labelled peptidoglycan and RNA cargo intracellularly (**Figure 4**). Considering that *B. fragilis* bacteria are non-invasive and do not readily secrete immunostimulatory effector molecules *via* a known secretion system (57, 58), OMVs are emerging as a novel secretion mechanism utilized by *B. fragilis* to deliver immunostimulatory cargo into the cytoplasm of host epithelial cells. Consistent with our findings, a previous study reported the ability of OMVs produced by the closely related *Bacteroides thetaiotaomicron* to enter intestinal epithelial cells using both Caco-2 cells and small intestinal organoid models of OMV entry (59). The entry of *B. fragilis* OMVs into intestinal epithelial cells has not been well described, however previous studies have reported the uptake of *B. fragilis* OMVs by host dendritic cells *ex vivo*, which was thought to facilitate the activation of the cytoplasmic NOD2 immune receptor (22, 25). Additionally, OMVs produced by commensal and probiotic strains of *Escherichia coli* were also found to enter intestinal epithelial cells, whereby fluorescent labelling of their peptidoglycan cargo demonstrated the intracellular delivery of peptidoglycan and the subsequent activation of NOD1-dependent immune responses (21). In contrast to the limited studies reporting the delivery of commensal-derived peptidoglycan into host cells *via* BMVs, there are numerous studies reporting the ability of BMVs produced by pathogens including *H. pylori*, *Vibrio cholerae* and *Aggregatibacter actinomycetemcomitans* to contain peptidoglycan and induce the activation of NOD1- or NOD2-dependent immune responses upon their entry into host epithelial cells (5, 42, 60–62). The BMV-mediated delivery of bacterial RNA to host cells has been also observed for pathogen-derived BMVs (37, 44, 48), resulting in the activation of intracellular detectors of microbial RNA, TLR7 and TLR8 (6, 40), but this has not been characterized in the context of commensal-derived BMVs. Therefore, our findings identify that *B. fragilis* can deliver bacterial RNA to host epithelial cells *via* OMVs and suggests the possibility that other commensal organisms may also be capable of potentially delivering bacterial-derived RNA into host cells *via* this mechanism, and this forms the basis of future studies. Collectively, these findings demonstrate that *B. fragilis* OMVs enter intestinal epithelial cells to deliver peptidoglycan and RNA cargo intracellularly, resulting in the activation of their respective cytoplasmic PRRs.

Having shown that *B. fragilis* OMVs are enriched in peptidoglycan (**Figure 2**), as well as their ability to deliver this cargo intracellularly (**Figure 4**), we demonstrated that *B. fragilis* OMVs can activate NOD1, the intracellular receptor for Gram-negative peptidoglycan (**Figure 5A**). However, in contrast to a previous study reporting that *B. fragilis* OMVs activated NOD2 following phagocytosis by dendritic cells (25), *B. fragilis* OMVs did not activate NOD2 in our epithelial HEK-Blue cell model of PRR activation. This may be explained by the differences in OMV entry between phagocytic dendritic cells compared to non-phagocytic epithelial cells (63), in addition to the increased expression of NOD2 by cells of myeloid origin (64). Furthermore, *B. fragilis* OMVs activated the intracellular RNA receptor, TLR7, but did not activate TLR8 which can also detect microbial RNA. Whilst human TLR7 and TLR8 are both responsible for the detection of single-stranded RNA compounds, and can be activated by RNA delivered by *S. aureus* MVs into host epithelial cells (6), evidence suggests that TLR7 may have greater ligand sensitivity than TLR8 (65), thus providing a potential explanation as to why we did not see TLR8 activation by *B. fragilis* OMVs in our study. In addition, we did not observe TLR9 activation in response to stimulation with *B. fragilis* OMVs at any MOI examined in this study, which suggests that the amount of DNA delivered by *B. fragilis* OMVs may not have been sufficient to mediate TLR9 activation (35). We have previously shown that BMVs produced by different strains of a bacterial species vary in their amount of DNA and RNA cargo and therefore differ in their ability to activate their respective TLRs (35). Therefore, although we did not see activation of TLR8 and TLR9 by *B*.* fragilis* OMVs in our study, we cannot exclude the possibility that stimulation of these cells with an increased amount of OMVs, or with OMVs produced by a different *B. fragilis* strain that harbor a greater concentration of DNA and RNA, may activate these TLRs.

Most importantly, the findings of our study revealed that *B. fragilis* bacteria did not induce the activation of any intracellular receptor tested in this study, which may be due to the bacterium being unable to directly deliver their biological cargo intracellularly. Therefore, the ability of the commensal *B. fragilis* to produce OMVs that mediate the activation of intracellular receptors NOD1 and TLR7 enables *B. fragilis* to activate a broader range of immune receptors at the epithelium. Collectively these findings suggest that OMV secretion by *B. fragilis* is a novel mechanism used by this bacterium to increase their potential to mediate immune crosstalk at the intestinal epithelium.

Overall, our findings identify that *B. fragilis* OMVs are enriched in immunostimulatory cargo and can transport this cargo directly into host epithelial cells to preferentially activate host PRRs compared to their parent bacteria. Furthermore, whilst previous studies have recognized the immunomodulatory properties of commensal bacteria or their BMVs, this study compares and provides evidence of key differences in the abilities of *B. fragilis* OMVs and *B. fragilis* bacteria to activate host TLRs and NODs. Therefore, OMVs emerge as a novel secretion mechanism used by *B. fragilis* and potentially other non-invasive commensal bacteria to mediate TLR and NOD activation in epithelial cells. In this way, commensal-derived BMVs may directly contribute to immune activation or modulation at the intestinal mucosal surface. Further research elucidating the composition and ability of other commensal and microbiota-derived BMVs to selectively deliver immunogenic cargo, and to activate and signal *via* host innate immune receptors is needed to improve our understanding of their contribution to maintaining homeostasis in the gastrointestinal niche.

## Data availability statement

The original contributions presented in the study are included in the article/**Supplementary Material**. Further inquiries can be directed to the corresponding author.

## Author contributions

All authors performed the research. WG, EJ, AFH and MK-L wrote the manuscript. All authors contributed to the article and approved the submitted version.

## Funding

This work was supported by an Australian Research Council Discovery Grant (MK-L and AFH, Grant number DP190101655) and by the LIMS Bioimaging Platform (La Trobe University, Australia). MK-L is supported by a veski Inspiring Women Fellowship.

## Conflict of interest

The authors declare that the research was conducted in the absence of any commercial or financial relationships that could be construed as a potential conflict of interest.

## Publisher’s note

All claims expressed in this article are solely those of the authors and do not necessarily represent those of their affiliated organizations, or those of the publisher, the editors and the reviewers. Any product that may be evaluated in this article, or claim that may be made by its manufacturer, is not guaranteed or endorsed by the publisher.

## Supplementary material

Supplementary Figure 1 Live and heat-killed *B. fragilis* bacteria activate TLR2, but not TLR4, without reducing host cell viability. HEK-Blue **(A)** null cells as well as **(B)** TLR2 and **(C)** TLR4 expressing HEK-Blue cells were stimulated with an increasing dose of either live (closed circles) or heat-killed (open circles) *B. fragilis* bacteria for 18 hours. Triangles represent positive controls for each respective cell line. Data represents mean ± SEM of three biological replicates. ns = not significant, *p < 0.05 (Unpaired *t*-test). **(D)** HEK-Blue null cells as well as **(E)** TLR2 and **(F)** TLR4 expressing HEK-Blue cells were stimulated with either live or heat-killed *B. fragilis* bacteria (MOI 1,000) for 18 hours, and cell viability was measured using MTT Assay. Non-stimulated cells (NS) were used as a control. Data represents mean ± SEM of at least three biological replicates. ns = not significant (One-way ANOVA with Tukey’s multiple comparisons test).

Supplementary Figure 2 Live and heat-killed *B. fragilis* bacteria do not activate NOD1 or NOD2, and do not decrease the viability of NOD1-expressing cells. HEK-Blue cells expressing **(A)** NOD1 or **(B)** NOD2 were stimulated with an increasing dose of either live (closed circles) or heat-killed (open circles) *B. fragilis* bacteria for 18 hours. Triangles represent positive controls for each respective cell line. Data represents mean ± SEM of three biological replicates. ns = not significant (Unpaired *t*-test). **(C)** The viability of NOD1-expressing HEK-Blue cells following 18 hours stimulation with either live or heat-killed *B. fragilis* bacteria (MOI 1,000) was measured by MTT Assay. Non-stimulated cells (NS) were used as a control. Data represents mean ± SEM of four biological replicates. ns = not significant (One-way ANOVA with Tukey’s multiple comparisons test).

Supplementary Figure 3 Live and heat-killed *B. fragilis* bacteria do not activate TLR7, TLR8 or TLR9, and do not decrease the viability of TLR7-expressing cells. HEK-Blue cells expressing **(A)** TLR7, **(C)** TLR8 or **(D)** TLR9 were stimulated with an increasing dose of either live (closed circles) or heat-killed (open circles) *B. fragilis* bacteria for 18 hours. Triangles represent positive controls for each respective cell line. Data represents mean ± SEM of three biological replicates. ns = not significant, (Unpaired *t*-test). **(B)** The viability of TLR7-expressing HEK-Blue cells following 18 hours stimulation with either live or heat-killed *B. fragilis* bacteria (MOI 1,000) was measured by MTT Assay. Non-stimulated cells (NS) were used as a control. Data represents mean ± SEM of four biological replicates. ns = not significant (One-way ANOVA with Tukey’s multiple comparisons test).
