# Supplementary figures and images for: Bacteroides fragilis outer membrane vesicles preferentially activate innate immune receptors compared to their parent bacteria
Source: Front Immunol. 2022 Sep 20;13:970725. doi: 10.3389/fimmu.2022.970725 (PMC9592552; doi:10.3389/fimmu.2022.970725)

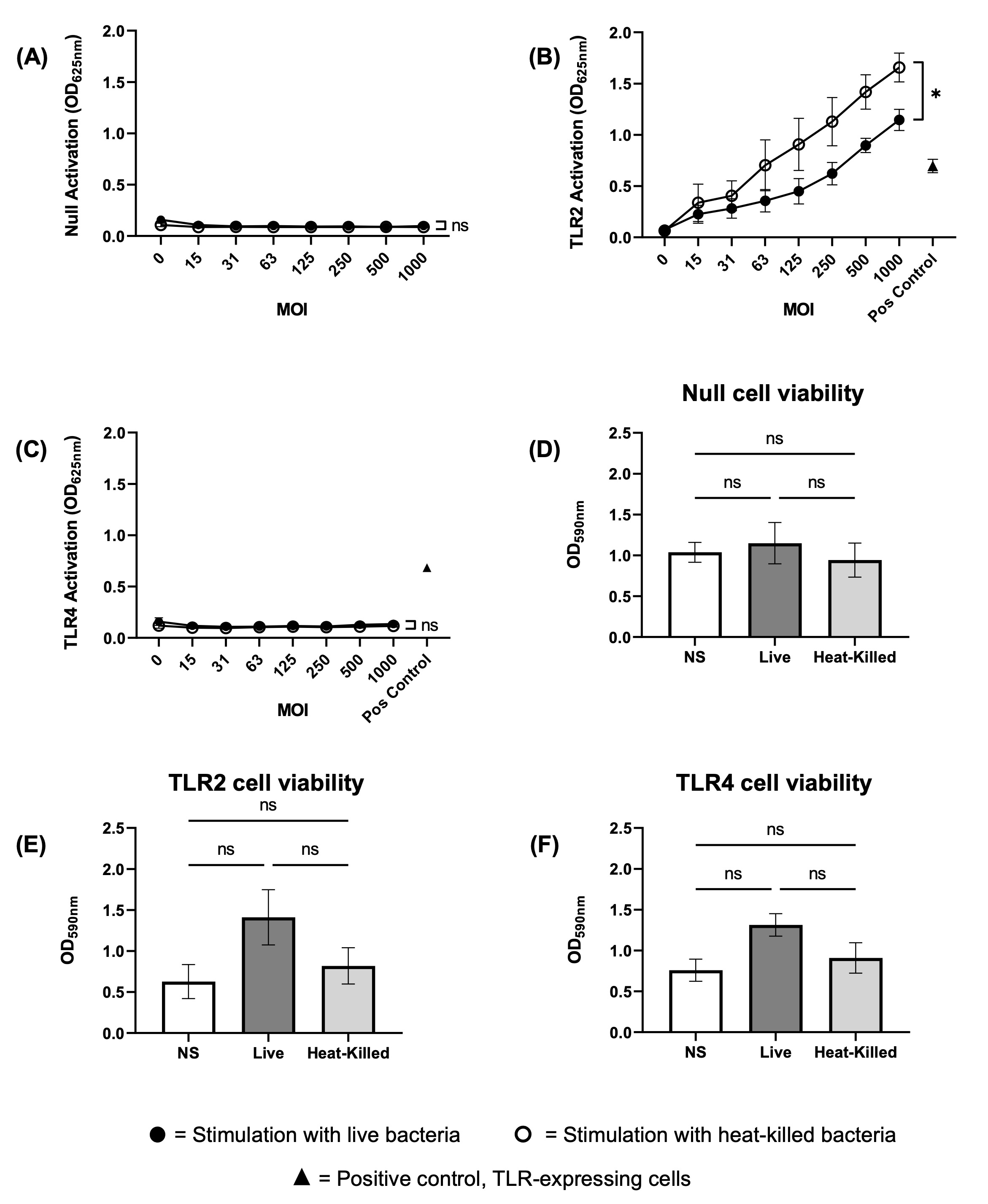

Supplement: Supplementary Figure 1 — Live and heat-killed B. fragilis bacteria activate TLR2, but not TLR4, without reducing host cell viability. HEK-Blue (A) null cells as well as (B) TLR2 and (C) TLR4 expressing HEK-Blue cells were stimulated with an increasing dose of either live (closed circles) or heat-killed (open circles) B. fragilis bacteria for 18 hours. Triangles represent positive controls for each respective cell line. Data represents mean ± SEM of three biological replicates. ns = not significant, *p < 0.05 (Unpaired t-test). (D) HEK-Blue null cells as well as (E) TLR2 and (F) TLR4 expressing HEK-Blue cells were stimulated with either live or heat-killed B. fragilis bacteria (MOI 1,000) for 18 hours, and cell viability was measured using MTT Assay. Non-stimulated cells (NS) were used as a control. Data represents mean ± SEM of at least three biological replicates. ns = not significant (One-way ANOVA with Tukey’s multiple comparisons test). [file Image_1.jpeg]

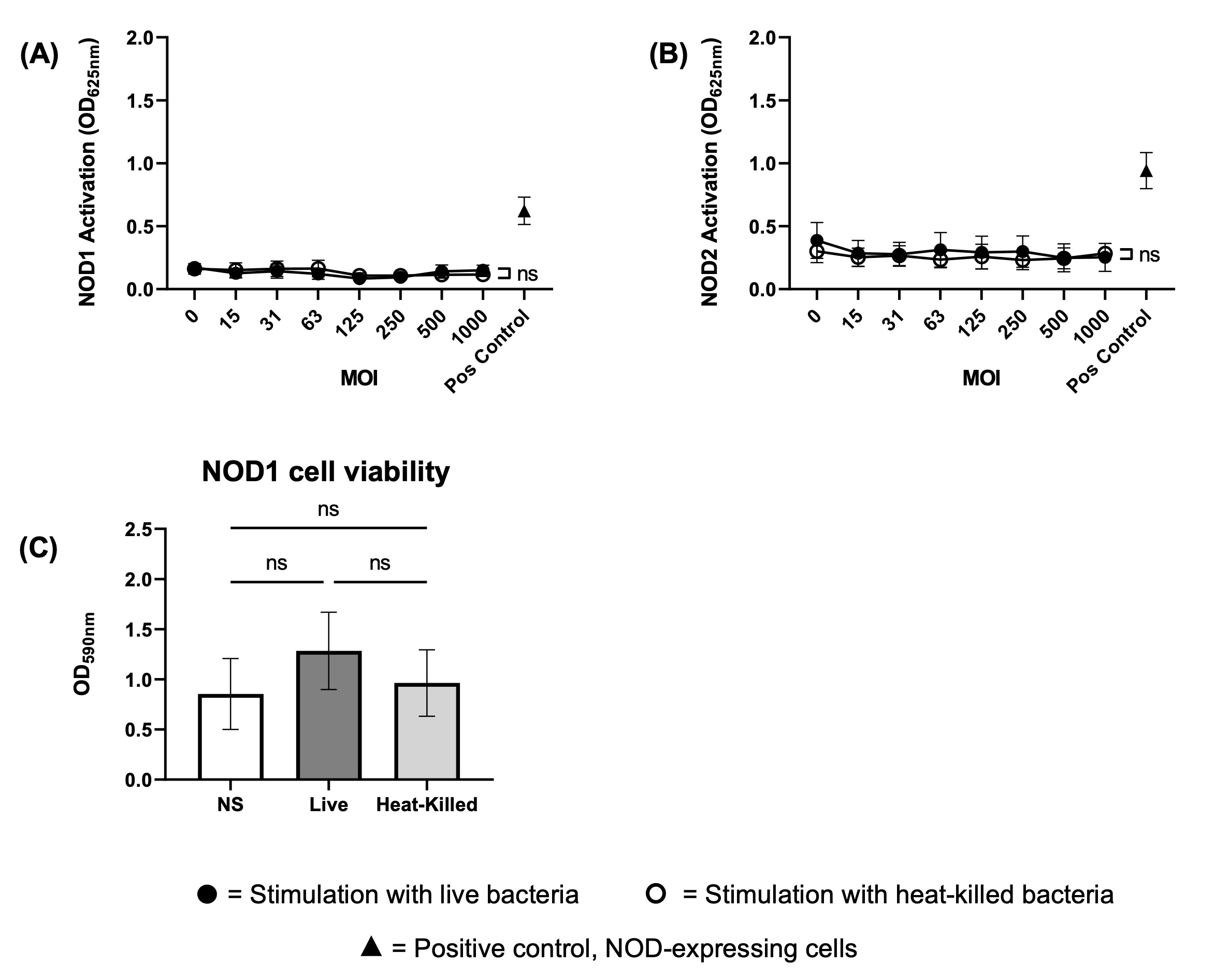

Supplement: Supplementary Figure 2 — Live and heat-killed B. fragilis bacteria do not activate NOD1 or NOD2, and do not decrease the viability of NOD1-expressing cells. HEK-Blue cells expressing (A) NOD1 or (B) NOD2 were stimulated with an increasing dose of either live (closed circles) or heat-killed (open circles) B. fragilis bacteria for 18 hours. Triangles represent positive controls for each respective cell line. Data represents mean ± SEM of three biological replicates. ns = not significant (Unpaired t-test). (C) The viability of NOD1-expressing HEK-Blue cells following 18 hours stimulation with either live or heat-killed B. fragilis bacteria (MOI 1,000) was measured by MTT Assay. Non-stimulated cells (NS) were used as a control. Data represents mean ± SEM of four biological replicates. ns = not significant (One-way ANOVA with Tukey’s multiple comparisons test). [file Image_2.jpeg]

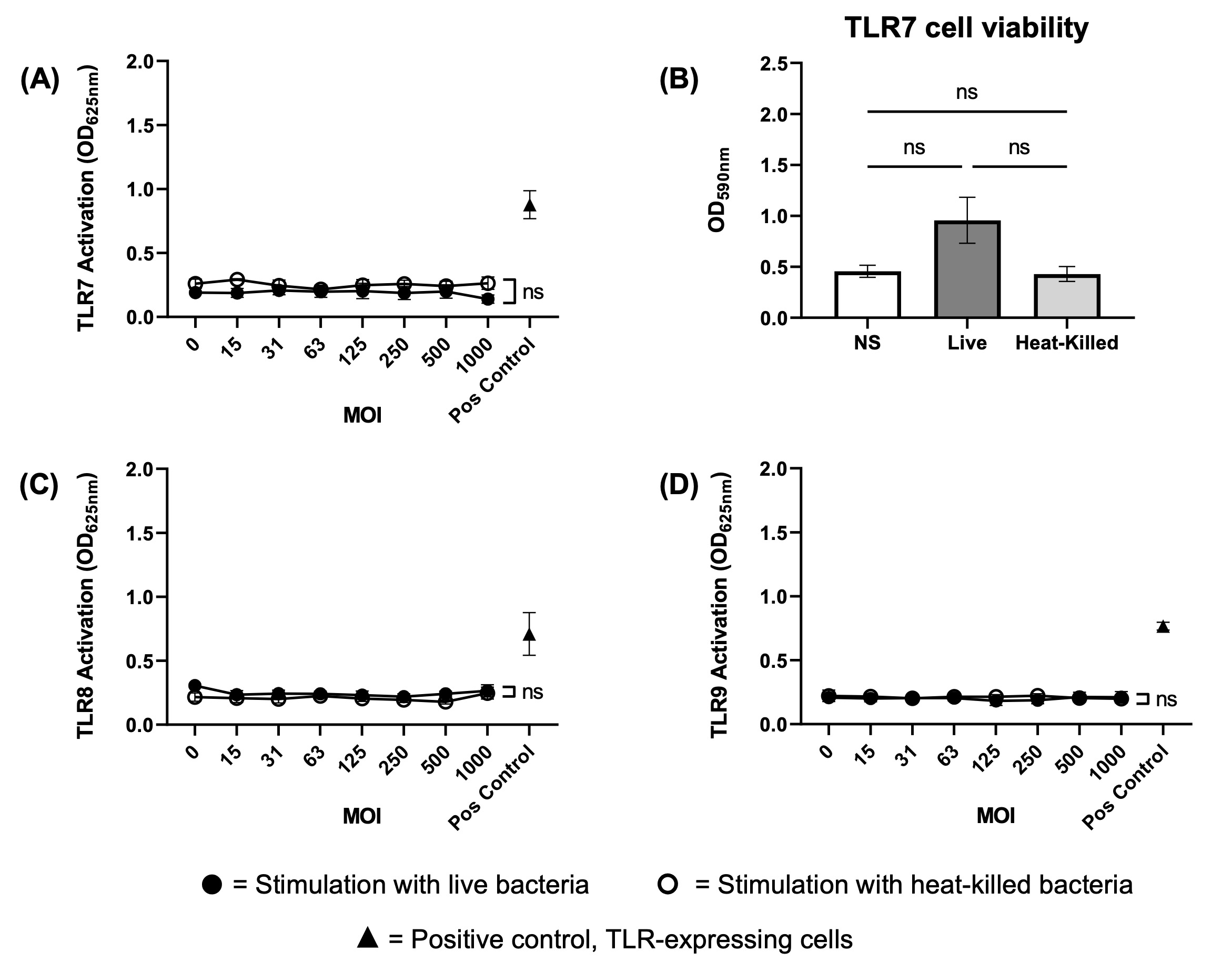

Supplement: Supplementary Figure 3 — Live and heat-killed B. fragilis bacteria do not activate TLR7, TLR8 or TLR9, and do not decrease the viability of TLR7-expressing cells. HEK-Blue cells expressing (A) TLR7, (C) TLR8 or (D) TLR9 were stimulated with an increasing dose of either live (closed circles) or heat-killed (open circles) B. fragilis bacteria for 18 hours. Triangles represent positive controls for each respective cell line. Data represents mean ± SEM of three biological replicates. ns = not significant, (Unpaired t-test). (B) The viability of TLR7-expressing HEK-Blue cells following 18 hours stimulation with either live or heat-killed B. fragilis bacteria (MOI 1,000) was measured by MTT Assay. Non-stimulated cells (NS) were used as a control. Data represents mean ± SEM of four biological replicates. ns = not significant (One-way ANOVA with Tukey’s multiple comparisons test). [file Image_3.jpeg]
